# Preliminary characterization of a novel β-agarase from *Thalassospira profundimonas*

**DOI:** 10.1186/s40064-016-2748-6

**Published:** 2016-07-15

**Authors:** Cheng Zeng, Longtao Zhang, Song Miao, Yi Zhang, Shaoxiao Zeng, Baodong Zheng

**Affiliations:** College of Food Science, Fujian Agriculture and Forestry University, Fuzhou, Fujian People’s Republic of China; Teagasc Food Research Centre, Moorepark, Fermoy, Co. Cork Ireland

**Keywords:** Agarase, Characterization, Purification, *Thalassospira profundimaris*

## Abstract

**Background:**

The objective of this study was to characterize the agarase from a newly isolated agarolytic bacterium *Thalassospira profundimaris* fst-13007.

**Results:**

Agarase-fst was purified to homogeneity which apparent molecular weight was 66.2 kDa. Its activity was optimal at 45 °C and pH 8 and was stable at pH 5–9 or 30–50 °C. Agarase-fst required Mn^2+^ for agarase activity and inhibition by Cu^2+^, Fe^3+^ and EDTA. Tests of hydrolysis pattern and substrate specificity, TLC analysis and mass spectrometry of the hydrolysis products revealed that it is an endo-type β-agarase hydrolyzing agarose into neoagarobiose, neoagarotetraose and neoagarohexaose. Results of MALDI-TOF-TOF/MS indicate that it lack of homology to previously identified proteins and present conserved domain of β-agarase.

**Conclusion:**

Agarase-fst from *T. profundimaris* fst-13007 was confirmed to be a novel endo-type β-agarase.

## Background

Agar, composed of agaropectin and agarose, is the main component in cell wall of the red algae. Oligosaccharides derived from agar have many useful properties, such as delayed starch degradation, bacterial growth inhibition, anticancer and antioxidation activities, etc. (Seok et al. 2012). Agarases are glycoside hydrolases (GHs) that catalyse the degrading of agarose, which is classified into β-agarase (EC 3.2.1.81), α-agarases (E.C.3.2.1.158), and β-porphyranases (EC 3.2.1.-) (Fu and Kim 2010). β-agarases hydrolyze agarose at the β-1,4 linkages into series of neoagarooligosaccharides (NAOS) with D-galactose at the reducing end. Nearly 50 agarases have been characterized, and most of them are β-agarases. α-agarases hydrolyze agarose at the α-1, 3 linkages into agarooligosaccharides with 3,6-anhydro-l-galactose at the reducing end (Potin et al. 1993). β-Porphyranases hydrolyze porphyrobiose at the β-1,4 linkages into series of porphyrooligosaccharides with D-galactose at the reducing end (Hehemann et al. 2010). A considerable number of agar-degrading enzymes from 34 microorganisms have been characterised (Chi et al. 2012). While, to the best of our knowledge, only β-agarase from *Pseudomonas atlantica*could be purchased in the market, such as from Sigma.

In this work, a novel agarase was firstly purified from a newly isolated agarolytic bacterium, *Thalassospira profundimaris*.

## Methods

### Materials

DEAE-Sepharose Fast Flow and Sephacryl S-100 (AKTA purifier) were purchased from GE Inc., Sweden. MALDI-TOF-TOF/MS (ABI 5800 plus) were purchased from Applied Biosystems, USA. MS Agilent LC1290-Triple-Q 6410 was purchased from Agilent Technologies, USA. Other chemicals were purchased from Sigma Inc., USA.

#### Isolation and identification of fst-13007

Seawater sample, collected from the offshore sea of Xiamen (northern latitude 24°27′, east longitude 118°04′, 2.7 % of salinity, 20.9 °C), China, were initially cultivated at 28 °C in a marine medium 2216 contained 1.50 % (w/v) agarose as the single carbon source. After incubation at 37 °C for 24–48 h, colonies that formed depressions on agarose plates were picked out and transferred onto new plates until the colony morphology was unchanged. The plates were stained using Lugol’s solution (5 g iodine crystals and 10 g KI in 100 ml distilled water) and agarase activity was visualized by zones of clearing. The colony on the plates showed the highest agarase activity was selected to cultivate for the following investigation. Its 16S rDNA was amplified, sequenced, and compared with the 16S rDNA sequences deposited in the GenBank database with the BLAST program.

### Enzyme assay

Agarase activity was measured by determining the amount the reducing sugar according to the DNS method by Miller (1959) with minor modification. Briefly, 50 μl enzymes were mixed with 950 μl Tris/HCl buffer (20 mM, pH 8, buffer A) containing 0.2 % (w/v) agarose as a substrate. After incubation at 45 °C for 30 min, the sample was mixed with 500 μl of 3,5-dinitrosalicylic acid reagent solution. The tubes were heated in boiling water for 10 min, and then cooled in an ice water bath. OD values were measured at 540 nm. The amount of enzyme that released 1 μmol of galactose per min under the assay conditions was defined as one unit (U) of agarase. D-galactose was used as a reference reducing sugar for preparing the standard curve.

### Purification

The strain fst-13007 was cultured in 1.5 l medium (NaCl 3 %(w/v), KCl 0.1 %(w/v), CaCl_2_ 0.01 %(w/v), MgSO_4_ 0.05 %(w/v), FeSO_4_ 0.002 %(w/v), Fe_2_(SO_4_)_3_ 0.001 %(w/v), tryptone 0.50 %(w/v), yeast extract 0.10 %(w/v), pH 8) supplemented with 0.2 % (w/v) agarose as the sole carbon source at 37 °C for 48 h. The purification was applied to 80 % (w/v) saturation (NH_4_)_2_SO_4_ precipitation, ultrafiltration, DEAE Fast Flow, and Sephacryl S-100 gel column sequentially. Fractions with agarase activity were collected and subjected to enzyme activity assay and electrophoresis (SDS-PAGE).

### Maldi-tof-tof/ms

The agarase band was excised from the SDS-PAGE staining gel. After enzymatic hydrolysis, the agarase was subjected to MALDI-TOF-TOF/MS analysis (ABI 5800 plus) to identify the purified protein. Both the MS and MS/MS data were integrated and processed with the GPS Explorer V3.6 software (Applied Biosystems, USA) with default parameters. Based on combined MS and MS/MS spectra, proteins were successfully identified based on 95 % or higher confidence interval of their scores in the MASCOT V2.3 search engine (Matrix Science Ltd., London, UK).

### Determination of enzymatic characteristics

The optimum temperature of agarase activity was determined in buffer A at different temperatures (20–60 °C). After pre-incubation at temperatures ranging from 20 to 60 °C for 1 h, the temperature stability of enzyme was determined under the defined optimal conditions (45 °C and pH 8). The optimum pH condition was determined at 45 °C using various buffers ranging from pH 3 to 11. For pH stability measurements, the agarase-fst was maintained at 45 °C for 1 h in these buffers before assaying. The effect of metal ions (1 mM), inhibitors (2 mM), and denaturing reagents (2 mM) on enzyme activity was assayed in buffer with various chemicals. All experiments were carried out a minimum of three times.

### Determination of substrate specificity

To determine the substrate specificity of agarase-fst, *p*-nitrophenyl-α-d-galactopyranoside and *p*–nitrophenyl-β-d-galactopyranoside were used as artificial chromogenic substrates for enzyme assaying (Chi et al. 2015). The reaction was carried out at 45 °C for 12 h and then stopped by the addition of 1 M Na_2_CO_3_. The release of *p*-nitrophenol from the hydrolysis of the artificial chromogenic substrate was measured using OD_420_.

### Hydrolysis pattern

Kinematic viscosity of the agarase was determined using a NDJ-79 Rotary viscometer (Shanghai Precision Instrument Co. China) and result was shown in Fig. 5.

### Enzymatic product analysis

The enzymatic product was analyzed by TLC and the molecular mass distribution was determined using LC–MS/MS. Results were shown in Figs. 6 and 7, respectively.

## Results and discussion

### Isolation and identification of fst-13007

**Fig. 1:**
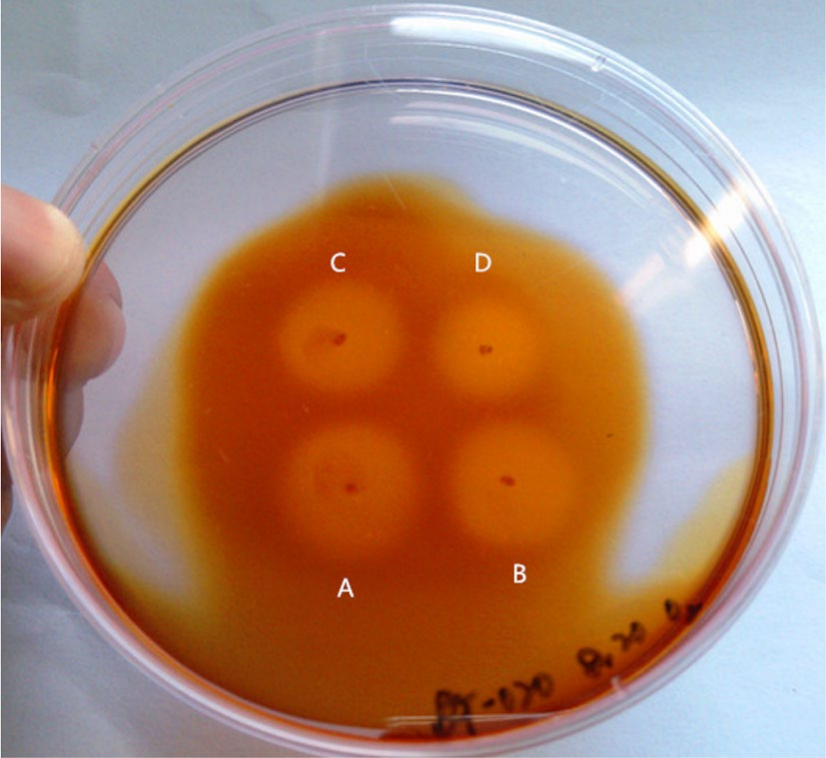
Lugol’s dyeing plate of agar-degrading strains

**Fig. 2:**
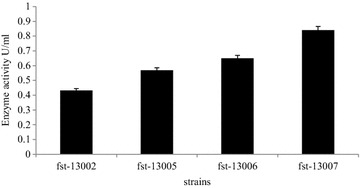
Agarase activity of four agar-degrading strains

In the primary screening experiment, 4 strains showed agarolytic activity (Fig. 1). The plates were stained using Lugol’s solution to check agarolytic ability. The strain A, strain B, strain C and strain D were named fst-13007, fst-13006, fst-13005 and fst-13002, respectively. Fst-13007 formed the biggest hole was selected and identified the highest enzyme activity. Its culture showed agarase activity at 0.84 U ml^−1^ (Fig. 2).

**Fig. 3:**
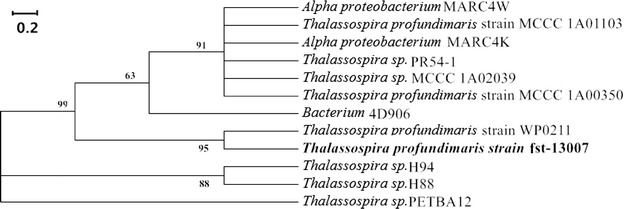
Phylogenetic analysis of strain fst-13007. The 16S rDNA sequence of strain fst-13007 was compared with the 16S rDNA sequences deposited in the GenBank database using the BLAST program. The phylogenetic tree of fst-13007 was made by the MEGA 5.0 and Clustalx software, using the neighbour-joining (NJ) tree method by analysing their 16S rDNA sequences. The *numbers at the nodes* show the bootstrap values obtained from 1000 resampling analyses

Its 16S rDNA gene sequence was deposited in GenBank with an Accession Number KU578316, showed 99 % similarity with *Thalassospira* sp. MCCC 1A00370. The phylogenic relationship with others chosen twelve bacterial species was reconstructed using the neighbour-joining (NJ) tree method. According to the topology, fst-13007 was grouped into the clade composed of annotated *T. profundimaris* species (Fig. 3). To the best of our knowledge, it is the first time of identifying a strain of *T. profundimaris* degrading agar, which has been deposited under the number M 2013708 in China Center for Type Culture Collection (CCTCC).

### Purification of agarase

**Fig. 4:**
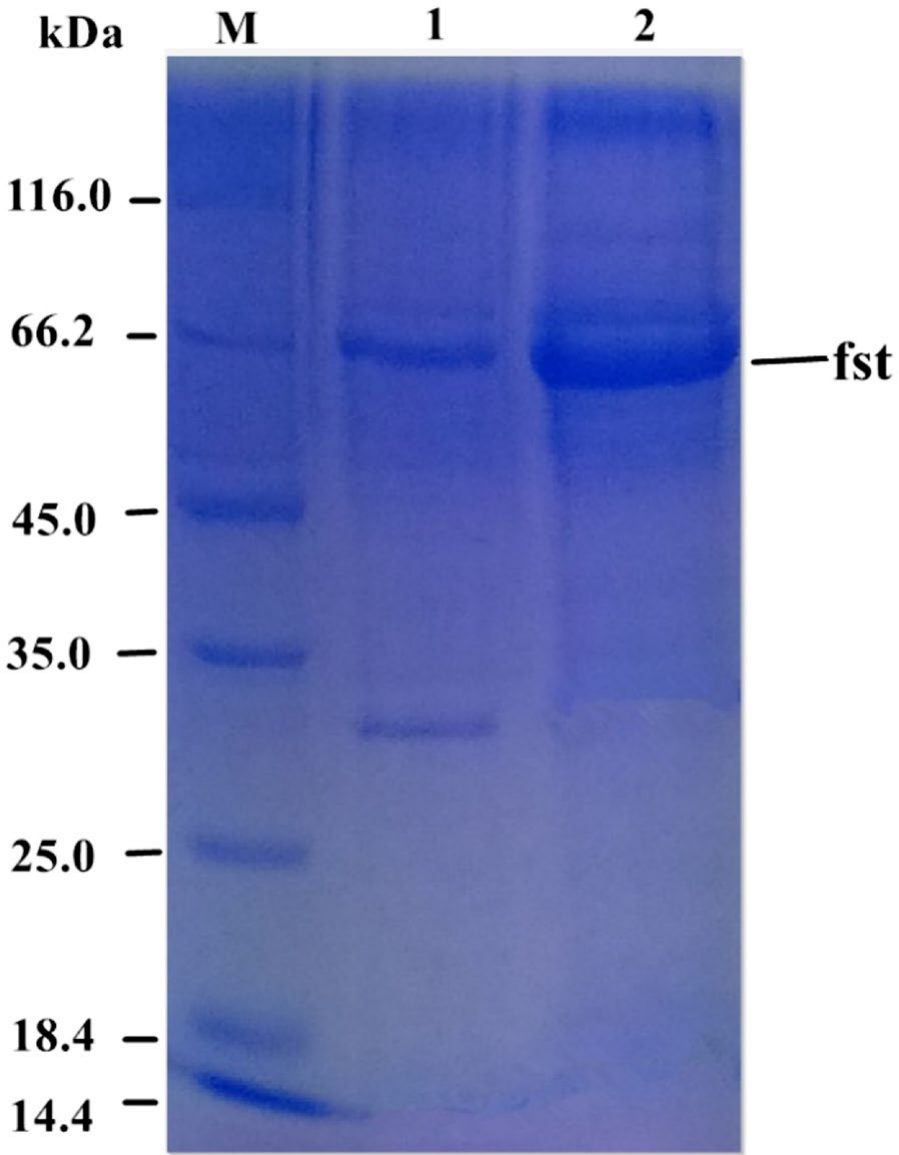
SDS-PAGE of the purified agarase-fst. *Lane M* Standard molecular mass markers; *Lane 1* agarase-fst after DEAE-Sepharose fast flow; *Lane 2*, agarase-fst after Sephacry1 S-100

The purification of agarase was summarized in Table 1. SDS-PAGE (Fig. 4) revealed that it was successfully purified and appeared on the gel as a single protein band after Sephacry1 S-100. It was purified 18.97-fold with a yield of 13.87 %. The specific activity of purified agarase was 1418 U mg ^−1^. Its molecular weight was approximately 66.2 kDa (Fig. 4).

**Table 1:** Purification of agarase-fst from *T. profundimaris* fst-13007

Purification step	Total protein (mg)	Total activity (U)	Specific activity (U mg^−1^)	Purification fold	Yield (%)
Crude enzyme solution	55.31	4134	74.74	1	100
(NH_4_)_2_SO_4_ precipitation	32.23	3903	121.08	1.62	94.4
DEAE FF	2.10	674	321	4.29	16.3
Sephacryl S-100	0.40	573	1418	18.97	13.87

After 20 min centrifugation at 12,000*g* and filtration through a 0.45 μm membrane, crude agarase-fst from the culture broth was precipitated by 80 % (w/v) ammonium sulfate saturation and left to stand overnight. After 20 min of centrifugation at 12,000 *g*, the precipitate was resuspended in 20 mM Tris/HCl buffer (pH 7.5, buffer B) and dialyzed against the same buffer overnight. The dialyzed section was ultrafiltrated through an ultrafilter (5000–10,000 kDa, transmembrane pressure of 0.5 bars). The active fractions were collected and applied onto a DEAE Sepharose Fast Flow (1.6 cm × 10 cm; GE Healthcare) equilibrated with buffer B. The bound proteins were eluted with gradient of 0–1 M NaCl in the same buffer. The flow rate was maintained at 1 ml min ^−1^. Active fractions were loaded onto Sephacryl S-100 gel column (1.6 cm × 60 cm; GE Healthcare) that had previously been equilibrated with buffer B. The enzyme was eluted with same buffer, at a flow rate of 1 ml min ^−1^. Unless stated, all purification procedures were performed at 4 °C

### Maldi-tof-tof/ms

**Fig. 5:**
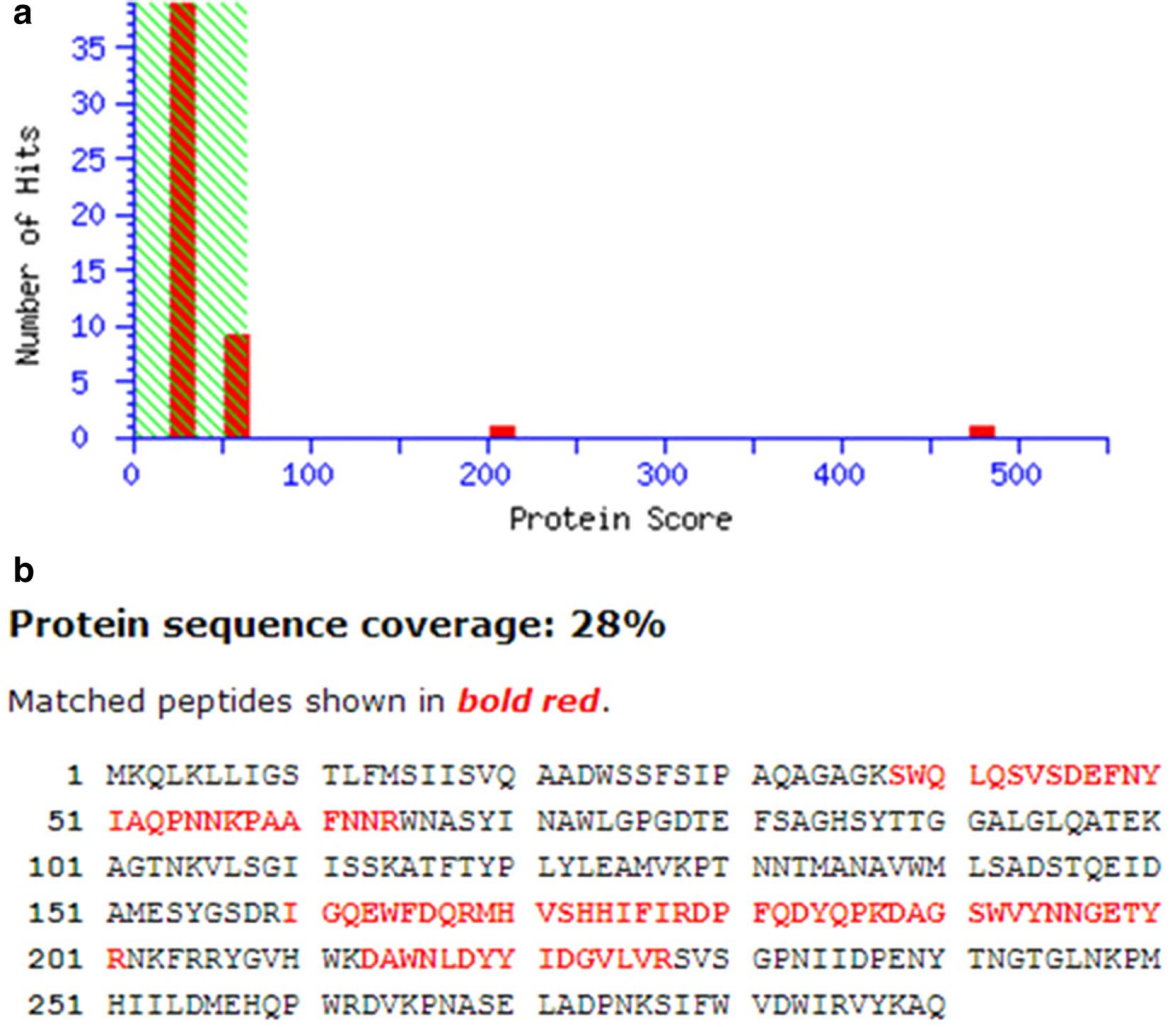
Mascot Score Histogram and Protein sequence coverage of agarase-fst. **a** Mascot Score Histogram of agarase-fst. Ions score is −10 × Log (p), where p is the probability that the observed match is a random event. Individual ions scores >64 indicate identity or extensive homology (p < 0.05). Protein scores are derived from ions scores as a non-probabilistic basis for ranking protein hits. **b** Protein sequence coverage of agarase-fst compared with agarase from *Pseudoalteromonas atlantica* (gi|1220461). Matched peptides shown in *red bold*

According to the mascot searching results in the database NCBInr 20151125 (77306371 sequences; 28104191422 residues), the protein coverages of agarases were analyzed and shown in Fig. 5. For agarase-fst, the protein score of β-agarase from *Pseudoalteromonas atlantica* (AAA91888, available in NCBI) was 480 (shown in Fig. 5a), which indicated that the agarase-fst had the extensive homology with β-agarase (AAA91888)(Oh et al. 2010). Its protein sequence coverage was 28 % against β-agarase (AAA91888) (Fig. 5b, matched peptides shown in red bold), which possesses the conserved domain (cd02178) of β-agarase belonging to GH16. Agarases were divided into four distinct families of glycoside hydrolases (GH), GH16, GH50, GH86 and GH118 according to the amino-acid sequence similarity. The results suggest the purified agarase-fst was a novel β-agarase, could be inferred belonging to GH16 family.

### Effect of temperature and pH on agarase activities

**Fig. 6:**
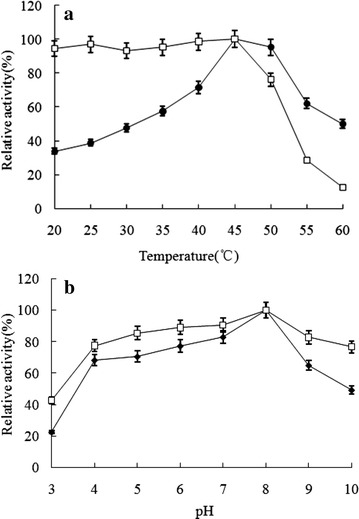
Effects of temperature and pH on the activity and stability of agarase-fst. **a** The effect of temperature. The agarase activity assaying was performed at pH 8 (20 mM Tris/HCl) with DNS method at different temperatures (20–60 °C). The maximum value (1.04 U ml^−1^) observed at 45 °C was considered 100 %. Thermostability of the enzyme was determined at 45 °C after preincubated at different temperatures (20–60 °C) for 1 h. The highest activity obtained was taken to be 100 %. Filled circles, optimum temperature; open squares, thermostability. **b** The effect of pH. The agarase activity assaying was performed at 45 °C at different pH conditions. The highest activity (0.96 U ml^−1^) obtained at pH 8 was considered 100 %. 20 mM sodium acetate buffer (pH 3–6), 20 mM Tris/HClbuffer (pH 7–9) and 20 mM glycine–NaOH buffer (pH 10). *Filled circles*, optimum pH; *open squares* pH stability

The temperature dependence of the agarase-fst activity reaches maximum at 45 °C. It was stable up to 45 °C (95 % activity remaining) and retained more than 70 % activity at 50 °C, but was inactivated after 1 h heat treatment at 60 °C (over 10 % remaining) (Fig. 6a). The agarase activity was maximized at pH 8 and remained active at pH values ranging from 4 to 10 (over 49 % remaining) (Fig. 6b), showing good pH stability in neural, mild acid and mild alkali conditions.

### Effects of chemicals on agarase activities

As shown in Table 2, the agarase was strongly inhibited by Cu^2+^, Fe^3+^ and EDTA. In contrast, it was a little activated by Mn^2+^ and Dithiothreitol (DTT). DTT could enhance the activity of agarase which indicated the possible existence of thiol at the catalytic site, as the reducing reagent could protect thiol from being oxidized to a disulfide bond (Fu et al. 2008).

**Table 2:** Effect of chemicals on the relative activities of agarase-fst

Chemicals	Relative activity (%)
Metal ions (1 mM)	
None	100
Ca^2+^	92.94 ± 1.65
Na^+^	93.29 ± 0.57
Mg^2+^	95.56 ± 1.01
Cu^2+^	43.77 ± 1.34
Fe^3+^	31 ± 0.38
Mn^2+^	162.54 ± 2.85
K^+^	97.35 ± 0.93
Other reagents (2 mM)	
SDS	94.20 ± 2.04
EDTA	86.88 ± 1.67
DTT	134.67 ± 1.13
Urea	100.42 ± 0.62

The reaction was conducted in 20 mM Tris/HCl (pH 8) at 45 °C with 0.2 % (w/v) agarose. Metal ions (CaCl_2_, MgCl_2_, CuSO_4_, FeCl_3_, MnCl_2_, NaCl and KCl) were added to the reaction mixture to a final concentration of 1 mM in the absence of 1 mM EDTA. While, the inhibitors and denaturing reagents (EDTA, SDS, DTT, and urea) were added to the reaction mixture to a final concentration of 2 mM. Various types of reagents were measured under control blank samples. Relative activity was defined as the proportion of agarase activity for each treatment relative to agarase activity with no reagent. The agarase activity without metal and EDTA was taken to be 100 % (0.93 U ml^−1^). Relative activity (%): average relative activity ± SD. After preincubation with the chemicals for 10 min, the enzyme assay was performed

### Hydrolysis pattern

**Fig. 7:**
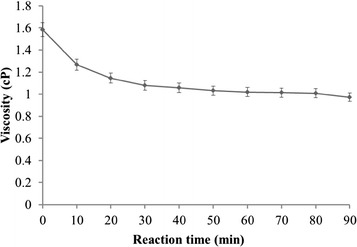
Viscosity changes of the reaction mixture. The hydrolysis reactions were conducted at 45 °C in 20 mM Tris/HClbuffer (pH 8) containing 0.5 % (w/v) agarose. Kinematic viscosity was determined every 10 min from 0 to 90 min with a NDJ-79 Rotary Viscometer at 45 °C. The test was repeated three times

The viscosity of reaction mixture significantly decreased from the first 30 min and gradually decreased until the end of the reaction, showing that the agarase-fst is an endo-type agarase (Fig. 7).

The test of Substrate specificity of agarase-fst showed strong hydrolysis activity (OD_540_ = 0.87) toward *p*-nitrophenyl-β-d-galactopyranoside, but negligible activity (OD_540_ = 0.06) toward *p*-nitrophenyl-α-d-galactopyranoside, indicating that it cleavage the β-linkage but not the α-linkage. Results suggest that agarase-fst is a β-agarase (Chi et al. 2015), which is consistent with the MALDI-TOF-TOF/MS results stated above.

### Analysis of hydrolysis products

**Fig. 8:**
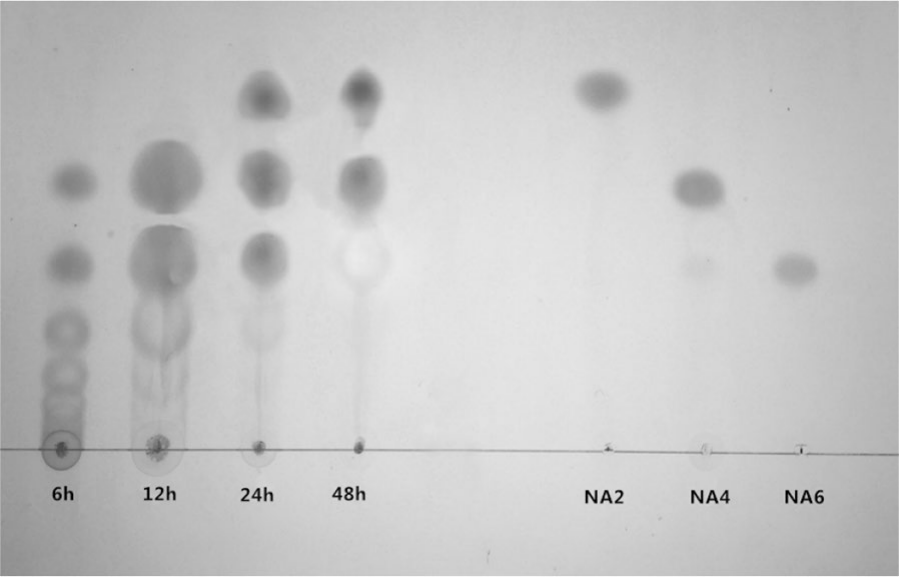
TLC analysis of agarose degradation by agarase-fst. The hydrolysis reactions were performed at 45 °C for 48 h in 20 mM Tris/HClbuffer (pH 8) containing 0.2 % (w/v) agarase. Aliquots of the reaction mixture (10 μl) at 6, 12, 24, and 48 h were spotted onto silica gel 60 plates (Merck, Germany) and developed using a solvent system composed of n-butanol, acetic acid and water (2:1:1, v/v/v). The resulting spots were visualized by spraying with 10 % (v/v) H_2_SO_4_ and heating at 95 °C for 10 min. Neoagarobiose (NA2), neoagarotetraose (NA4) and neoagarohexaose (NA6) were used as standards

TLC analysis results were shown in Fig. 8. In the beginning of the reaction, the predominant products were NA4and NA6. After 24 h, the NA2 began to be observed. It could be inferred that various lengths of NAOS longer than NA6 were continuously hydrolysed and NAOS with short chain was gradually accumulated. After 48 h, two major spots (NA2 and NA4) and one negligible spot (NA6) were observed. Up to now, this is the third agarase hydrolyzing agar to NA2 and NA4and NA6, except for another two agarases from Zobellia galactanivorans (Jam et al. 2005) and Microbulbifer Thermotolerans JAMB-A94 (Ohta et al. 2004) respectively.

**Fig. 9:**
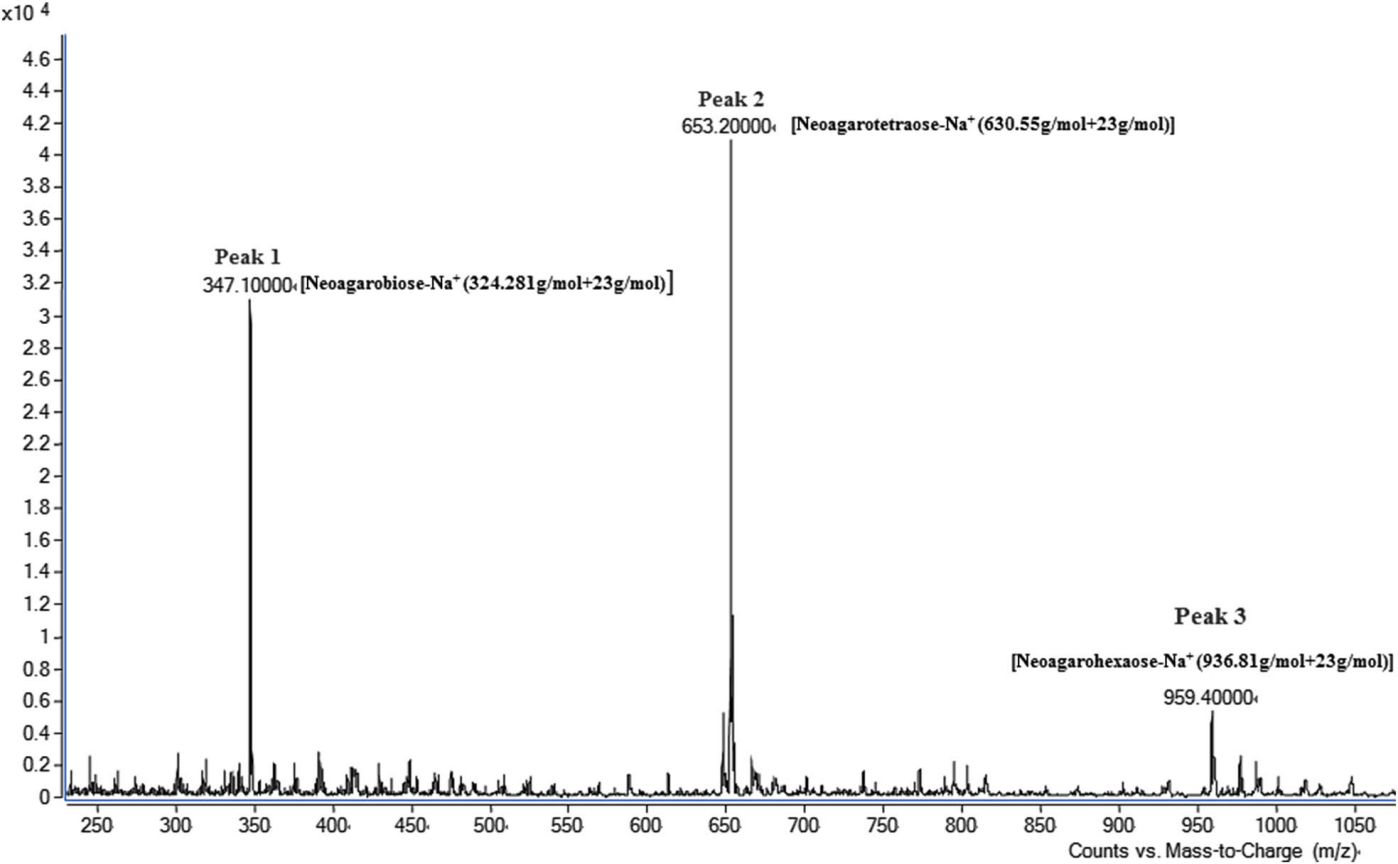
LC-MS/MS spectrum of the enzymatic products of agarose resulting from agarase-fst. Samples were resuspended with 5 µl 0.1 % (w/v) TFA followed by mixing (1:1, v/v) with a matrix consisting of a saturated solution of α-cyano-4-hydroxy-trans-cinnamic acid in 50 % (w/v) ACN, 0.1 % (w/v) TFA. And 1 μl mixture was spotted on a stainless steel sample target plate. The molecular mass distribution was determined using MS Agilent LC1290-Triple-Q 6410 (Agilent Technologies, USA). Ionization was achieved by atmospheric pressure electrospray ionization in the positive mode with an ESI capillary voltage of 4 kV. The mass spectra were recorded in the range of *m/z* 50 and1500

To determine the molecular mass distribution, the hydrolysis products were analysed by LC–MS/MS. As shown in Fig. 9, peak 1 was revealed to have a *m/z* ratio of 347 (M + Na)^+^, which was applicable to NA2, and peak 2 was identified as NA4 [*m/z* = 653 (M + Na)^+^]. The peak 3 with molecular weight of 959 Da corresponded to the molecules of (M + Na)^+^, which was attributed to NA6 (936 Da). According to the relative peak intensities, hydrolysis products contained 40 % NA2, 53 % NA4 and 7 % NA6.

## Conclusions

An external agarase-fst was purified from *T. profundimaris* and characterized. Based on the biochemical characteristics of the enzyme and lack of homology to previously identified proteins, it can be concluded that the agarase-fst is a novel endo-type β-agarase hydrolyzing agarose into NA2, NA4 and NA6. The bacterium and the agarase will be a new resource for the high value-added product development in the agar industry.
